# Lost in Translation: Interprofessional Variability in Understanding Pro Re Nata (PRN) Prescribing in Geriatric Practice—A Prospective Observational Study

**DOI:** 10.3390/geriatrics11040103

**Published:** 2026-08-11

**Authors:** Pauline Ripoche, Thomas Rodier, Marine Grangé, Dany Vythilingum, Zohra Mekerta, Aude Tarré, Patrick Hindlet, Laurent Lechowski, Hugues Michelon

**Affiliations:** 1 Service de Pharmacie, Hôpitaux Sainte-Périne—Raymond Poincaré, Assistance Publique-Hôpitaux de Paris (AP-HP), Université Paris-Saclay, 75016 Paris, France; pauline.ripoche@aphp.fr (P.R.); thomas.rodier@aphp.fr (T.R.); aude.tarre@aphp.fr (A.T.); patrick.hindlet@aphp.fr (P.H.); 2 Department of Clinical Pharmacy, Faculty of Pharmacy, University of Paris-Saclay, 91100 Orsay, France; 3 Centre de Recherche en Épidémiologie et Santé des Populations (CESP), Equipe Epidémiologie Clinique et Pharmaco-Épidémiologie, Université Paris-Saclay, INSERM U1018, 94800 Villejuif, France; 4 Service de Médecine des Aînés, Hôpital Sainte-Périne, Assistance Publique-Hôpitaux de Paris (AP-HP), Université Paris-Saclay, 75016 Paris, France; marine.grange@aphp.fr (M.G.); dany.vythilingum@aphp.fr (D.V.); zohra.mekerta@aphp.fr (Z.M.); laurent.lechowski@aphp.fr (L.L.)

**Keywords:** Pro Re Nata, drug prescription, perception, patient safety, inpatient, geriatrics

## Abstract

**Background/Objectives:** Medication safety is a critical concern in older adults due to age-related vulnerability, multimorbidity, and polypharmacy. Pro Re Nata (PRN, “as needed”) prescribing is common in geriatric care but may be interpreted inconsistently across healthcare professionals, potentially increasing the risk of medication errors. This study aimed to evaluate PRN prescribing patterns in hospitalized older adults and assess interprofessional perceptions of the clarity and appropriateness of PRN prescribing for conditions. **Methods:** We conducted a prospective, single-day observational study in a French geriatric university hospital, including all patients aged ≥65 years with at least one medication prescription. PRN prescriptions were extracted from electronic medical records and independently assessed by a pharmacist, nurse, and physician for clarity and appropriateness. Descriptive statistics summarized PRN use, chi-square tests assessed interprofessional differences, and multivariate logistic regression identified predictors of appropriate PRN wording. **Results:** Among 316 patients, 1035 PRN prescriptions were analyzed, representing 24.5% of all medication entries. Only 51.3% (n = 531) were deemed appropriate by all evaluators. Significant differences in interpretation were observed across professional groups (*p* < 0.001 for most pairwise comparisons). PRN prescriptions were most frequent in long-term care units and primarily involved gastrointestinal agents, analgesics, and psychotropic medications. Multivariate analysis showed that hospitalization in long-term care (OR = 4.17; 95% CI 2.48–7.03) or rehabilitation units (OR = 2.07; 95% CI 1.22–3.53) with a higher number of prescriptions administered under a therapeutic protocol PRN (OR = 2.27; 95% CI 1.39–3.63) were independently associated with appropriate wording, while a higher total number of PRN prescriptions reduced appropriateness (OR = 0.87; 95% CI 0.82–0.94). **Conclusions:** PRN prescribing is frequent in hospitalized older adults and often involves high-risk or potentially inappropriate medications. Substantial interprofessional variability in the interpretation of PRN medication wording requires appropriate, standardized, and clinical PRN guidelines to improve medication safety and reduce potential adverse events in geriatric practice.

## 1. Introduction

Ensuring medication safety and reducing drug-related events are major public health priorities, particularly in older adults [1,2,3]. Advanced age, multimorbidity, and polypharmacy substantially increase the risk of adverse drug events and may alter the benefit–risk balance of medications. Polypharmacy itself represents an additional risk factor, as potential adverse effects and drug–drug interactions may accumulate. For each additional medication added to a prescription, the probability of experiencing a drug-related adverse event increases by 12% to 28% in older populations [4]. In addition to the overall burden of polypharmacy, medication-related problems in older adults are frequently associated with potentially inappropriate medications, prescribing complexity, and administration errors. PRN medications may represent an additional challenge because their use depends on clinical assessment at the time of administration and on the clarity of prescribing instructions. In nursing home populations, PRN medications have been reported to account for approximately 10–20% of all prescribed medications, with psychotropic drugs and analgesics among the most frequently involved classes [5,6]. Improving the safety and appropriateness of prescribing is therefore essential to limit—or even prevent—the harmful consequences of iatrogenesis in frail and high-risk older patients.

Pro Re Nata (PRN), or conditional prescribing (“as needed”), is a common practice in geriatric care, both in community and hospital settings, with reported prevalence ranging from 28% to 93% depending on the study [5,6,7]. PRN prescriptions frequently involve potentially inappropriate medications, including psychotropic drugs such as antipsychotics, anxiolytics, sedatives, hypnotics, and analgesics, as well as gastrointestinal agents [5,6,7,8]. As such, PRN prescribing represents a potentially high-risk prescribing practice, particularly in older adults. PRN prescribing is defined as the prescription of a medication intended to be administered only when a predefined clinical indication or symptom is present. Unlike routinely prescribed medications, PRN administration requires an individual clinical assessment before each dose. In routine clinical practice, nurses play a central role in this process by assessing the patient’s condition, symptoms, and need for medication whenever a patient requests treatment or a clinical need is identified, and by determining whether the prescribed condition for PRN administration has been met, in accordance with the prescriber’s instructions [9,10,11]. In France, according to the Direction de l’Hospitalisation et de l’Organisation des Soins, this type of prescription must be clearly structured, defined, and validated in collaboration with healthcare professionals, and integrated into a broader policy of appropriate medication use, prevention of iatrogenesis, and quality improvement [11]. Each PRN prescription should specify the drug name, route of administration, dosage, and duration of validity. The triggering conditions must be clearly defined and unambiguous to ensure safe administration and to prevent errors arising from differences in interpretation among healthcare professionals (physicians, pharmacists, and nurses) [11]. However, depending on how the condition is phrased, the decision to administer a PRN medication may be difficult to interpret, as it can rely on the perception or subjective judgment of the healthcare professional. This interpretation may differ from the prescriber’s and may contribute to medication-related adverse events [12,13,14]. Although the prevalence and safety of PRN prescribing have been investigated previously [5,14], little is known about how the wording of PRN prescriptions is interpreted by various healthcare professionals involved in medication management. Understanding this interprofessional variability is essential, as discrepancies in interpretation may contribute to medication errors and compromise patient safety. In France, limited data are currently available regarding the prevalence, benefits, and risks of PRN medications, particularly in older adults. While PRN prescribing may allow flexibility and timely symptom management, it requires clear, understandable, and clinically appropriate conditions to minimize medication errors—such as overtreatment—leading to adverse events or undertreatment resulting in missed therapeutic opportunities. The objective of this study was therefore to evaluate PRN prescribing in hospitalized older patients and to compare healthcare professionals’ perceptions of the clarity and appropriateness of PRN prescription wording to better understand the potential risks of administration-related errors.

## 2. Materials and Methods

### 2.1. Design and Setting

We conducted a prospective, cross-sectional “one-day” observational study assessing active medication prescriptions of older patients hospitalized at Sainte-Périne Hospital, a 343-bed geriatric university hospital (with 39 beds in an acute geriatric unit, 133 in a rehabilitation unit, and 210 in a long-term care unit) within the AP-HP Paris-Saclay University Hospital Group. The hospital includes three types of geriatric care settings with distinct clinical objectives and medication management contexts. The acute geriatric unit provides short-term hospitalization for older adults with acute medical conditions requiring diagnostic evaluation, stabilization, and rapid therapeutic adjustments. In this setting, medication prescriptions are frequently reviewed and modified according to changes in patients’ clinical status. The rehabilitation unit provides intermediate-duration care focused on functional recovery after an acute event, with medication management generally aimed at balancing ongoing treatments, rehabilitation goals, and evolving clinical needs. The long-term care units provide prolonged institutional care for older adults with chronic conditions, functional dependency, and frequent multimorbidity. In this setting, medication regimens are often more stable over time but may include a higher proportion of symptomatic treatments, including PRN medications for pain, behavioral symptoms, sleep disturbances, or gastrointestinal complaints.

One-day assessments were conducted in each geriatric care unit between 31 March and 28 April 2025. All active PRN prescriptions available in the computerized physician order entry system on the day of assessment were extracted and evaluated.

To address the potential bias in this study due to the case-mix design, we included patients from various types of care units. Additionally, the risk of bias associated with the single-day design was addressed by collecting data on a different day of the week for each care unit, aiming to capture a “snapshot” of real-life PRN prescribing practices in a geriatric hospital.

### 2.2. Population and Data Collection

All prescriptions for inpatients aged 65 years and older with at least one prescribed medication were included. Active prescription data were extracted from the computerized physician order entry system (Orbis^®^ v 08052200.17001.FR, Dedalus Healthcare, Artigues-près-Bordeaux, France) using a predefined anonymized Excel^®^ data collection form developed specifically for the study. The extracted variables included the total number of medication entries, PRN prescriptions, drug name (International Nonproprietary Name), wording of the conditional statement, route of administration, and duration of prescription. All collected data were anonymized before inclusion in the study database. The computerized physician order entry system (Orbis^®^, Dedalus) is our institutional electronic prescribing system used for medication management and represents the official source of active medication prescriptions. Data quality was ensured by performing systematic extraction with a clinical pharmacist according to predefined variables and by verifying the completeness of extracted prescription data before analysis.

### 2.3. Assessment of PRN Prescription Appropriateness

To assess interprofessional variability in the interpretation of PRN prescriptions, the wording of each PRN prescription was independently and blindly assessed by three evaluators: a physician, a nurse, and a clinical pharmacist. A physician and a nurse were recruited from each geriatric care setting: acute care (AC), rehabilitation care (RC), and long-term care (LTC). A single clinical pharmacist evaluated prescriptions from all three types of units. The aim of this assessment was to determine whether the wording of the PRN prescription provided sufficient information to support a safe and consistent administration decision in routine clinical practice. In this study, appropriateness referred exclusively to the adequacy and clarity of the PRN prescription’s wording and not to the clinical appropriateness of the prescribed medication itself. A PRN prescription was considered appropriate when the wording provided sufficient and explicit information on both the indication for administration and the clinical conditions required for safe medication administration according to the prescriber’s instructions. Appropriate wording could include objective clinical assessment criteria (e.g., pain intensity scales, blood pressure measurements, blood glucose values, bowel movement frequency, or other measurable clinical parameters) or sufficiently explicit clinical situations allowing safe and consistent decision-making. Conversely, a prescription was considered inappropriate when the wording was unclear, incomplete, or ambiguous and did not contain reliably identifiable administration criteria. This could potentially lead to variability in interpretation, under-treatment, over-treatment, or medication-related risks. No predefined assessment tool, checklist, or consensus framework was used, as this study’s purpose was to capture the spontaneous interpretation of PRN prescription wording by healthcare professionals in routine clinical practice. To remain as close as possible to real-life prescribing and administration conditions, evaluators were asked to rely on their usual professional judgment, taking into account their clinical experience, professional practice, and familiarity with evidence-based clinical guidelines and instructions for practice.

### 2.4. Covariates

For each PRN prescription, the following data were collected: total number of medication entries, number of PRN prescriptions, drug name (International Nonproprietary Name), wording of the conditional statement, and duration of prescription. All data were anonymized in the study database prior to analysis.

### 2.5. Ethics

Our research was conducted in accordance with the principles of the Declaration of Helsinki and with applicable national and institutional regulations. Ethical approval was not required for this single-center observational study, as it relied exclusively on healthcare professionals’ assessment of PRN prescription wording extracted from anonymized prescription data. In accordance with Article 35 of the General Data Protection Regulation (GDPR; Regulation (EU) 2016/679), a Privacy Impact Assessment was conducted and approved by the institutional Data Protection Department under reference number 202606091008.

### 2.6. Study Outcomes

The primary outcome was the proportion of PRN prescriptions considered appropriate. Secondary outcomes included interprofessional differences in the assessment of PRN prescription appropriateness and the identification of factors associated with appropriate PRN prescription wording.

### 2.7. Statistical Analysis

Prescription characteristics and categorical variables are presented as counts and percentages, and continuous variables are given as means with standard deviations (SDs). The PRN prescribing rate was calculated as the ratio of PRN medication entries to the total number of medication entries recorded for all hospitalized patients. Results are expressed as counts and proportions. An independent chi-square test was used to compare the interpretation of PRN prescriptions within the same professional group across different care settings. A *p*-value lower than 0.05 was considered statistically significant. As a supplementary analysis, inter-rater agreement between nurses, physicians, and clinical pharmacists was assessed using Cohen’s kappa coefficient for pairwise comparisons and Fleiss’ kappa coefficient for overall agreement.

Mixed-effects logistic regression models with a random intercept for patients were used to account for clustering of PRN prescriptions within individuals. Univariate logistic regression analyses were conducted to identify prescription characteristics associated with the appropriateness of PRN prescription wording. The following variables were tested in the univariate logistic regression models: type of care unit; total number of medications per prescription, prescription entries, PRN prescriptions, and prescriptions under a therapeutic protocol; non-opioid analgesics; and gastrointestinal medications and hypno-sedative medications. Dichotomized variables were also analyzed, including number of prescription entries (>5), PRN prescriptions (>2), and prescriptions under a therapeutic protocol (>1). Therapeutic classes representing more than 10% of PRN prescription entries were included as independent variables in the regression analyses. Any variable with a *p*-value <0.20 in the univariate analysis was included in the multivariate mixed-effects logistic regression model. A backward elimination approach was then applied to obtain the final model. We assessed collinearity using Pearson’s correlation coefficient (CP) and VIF (Variance Inflation Factor) methods. When CP > 0.8 or VIF > 2.5 between two variables, collinearity was present. In the final multivariate logistic regression model, c-Statistic was calculated. All analyses were performed using R statistical software (version 4.4.2).

## 3. Results

### 3.1. Characteristics and Rates of PRN Prescribing Medications

Among the 316 inpatients who received at least one prescribed medication, a total of 4206 prescription entries were analyzed. Of these, 1035 corresponded to PRN medications, representing an overall prevalence of 24.5% (Table 1 and Table 2). None of the PRN prescriptions included a documented stop date. PRN prescribing was predominantly observed in long-term care units (51.6%), where the highest rates were recorded compared with the other wards. During the study period, 99% of patients were administered at least one PRN medication. The mean number of PRN prescriptions per patient was 3.21 ± 1.77 in acute care, 3.44 ± 1.86 in long-term care, and 3.08 ± 1.37 in rehabilitation care. Medications targeting the gastrointestinal system (laxatives, antispasmodics, and antiemetics) were the most frequently prescribed PRN drugs (38.4%), followed by analgesics (32.9%) and psychotropic agents such as anxiolytic or hypnotic drugs (13.9%).

### 3.2. Perception of PRN Prescription Wording

Evaluating the understanding and appropriateness of wording in PRN prescriptions revealed significant differences in perceptions among healthcare professionals. Overall, the proportion of PRN prescriptions considered appropriate differed significantly across professional groups (Table 3 and Table 4). Only 51.3% (n = 531) were deemed appropriate by all healthcare professionals. Furthermore, insulin was the only medication prescribed PRN within the protocol, and 100% agreement was observed for protocolized PRN insulin prescriptions.

Among nurses, a significant difference was observed between acute care and long-term care settings (*p* < 0.001), as well as between long-term care and rehabilitation units (RC) (*p* = 0.022). In contrast, no significant difference was found between acute care and rehabilitation units (*p* = 0.31) nor between long-term care and rehabilitation units (*p* = 0.44). Comparisons between nurses and physicians within the same care settings also revealed significant discrepancies between perceptions. The proportion of PRN prescriptions rated as appropriate differed significantly between nurses and physicians in acute care (*p* < 0.001), long-term care (*p* < 0.001), and rehabilitation units (*p* < 0.001). Similarly, physicians’ assessments differed significantly across care settings, with marked differences between acute and long-term care (*p* < 0.001), acute and rehabilitation units (*p* < 0.001), and long-term and rehabilitation units (*p* < 0.001). The clinical pharmacist consistently demonstrated significantly different perceptions compared with both nurses and physicians across all care settings (all *p* < 0.001).

Overall, these findings indicate substantial interprofessional variability in interpreting PRN prescription instructions. With the exception of nurses in acute and rehabilitation units, nearly all pairwise comparisons revealed statistically significant differences, suggesting that the clarity of wording in PRN prescriptions is not interpreted uniformly across professional groups or clinical contexts. Inter-rater agreement analyses are presented in Supplementary Table S1. Pairwise Cohen’s kappa coefficients ranged from 0.17 to 0.49, indicating slight-to-moderate agreement between healthcare professionals. Overall agreement among nurses, physicians, and the clinical pharmacist was similar across all care settings, with Fleiss’ kappa coefficients of 0.28 in acute care, 0.33 in rehabilitation care, and 0.36 in long-term care. These findings are consistent with the chi-square analyses and support the substantial interprofessional variability found in the interpretation of conditional PRN prescriptions.

### 3.3. Predictors of Adapted PRN Prescription Wording

Factors associated with the appropriateness of wording in PRN prescriptions were analyzed using univariate analyses followed by multivariate logistic regression (Table 5). The multivariate model included the following covariates: care unit; number of prescription entries (>5), PRN prescriptions, and prescriptions under a therapeutic protocol (>1); non-opioid analgesics; gastrointestinal medications; and hypno-sedative medications.

In the multivariable logistic regression model, several factors remained independently associated with appropriate PRN wording (Table 5). Compared with acute geriatric care units, hospitalization in long-term care units was associated with a higher likelihood of appropriate PRN prescription wording (OR = 4.17; 95% CI [2.48–7.03]; *p* < 0.001). Similarly, hospitalization in a rehabilitation unit was associated with an increased odds ratio for appropriate wording (OR = 2.07; 95% CI [1.22–3.53]; *p* = 0.007). The total number of PRN medications was inversely associated with appropriateness (OR = 0.87; 95% CI [0.82–0.94]; *p* < 0.001. Conversely, PRN medications used as part of protocols (e.g., insulin protocols) were positively associated with appropriate wording (OR = 2.27; 95% CI [1.39–3.63]; *p* = 0.001). Regarding medication classes, gastrointestinal agents were associated with a higher likelihood of appropriate PRN prescription wording (OR = 4.37; 95% CI [3.22–5.94]; *p* <0.001). In contrast, hypno-sedative medications were strongly and inversely associated with appropriateness (OR = 0.05; 95% CI [0.02–0.11]; *p* < 0.001).

## 4. Discussion

### 4.1. Key Results and Interpretations

This prospective study highlights substantial challenges associated with PRN prescribing in geriatric populations. Nearly one-quarter of all medication entries corresponded to PRN prescriptions, and only half of these were considered appropriate across all professional groups. Most importantly, we observed marked interprofessional variability in the interpretation of wording for PRN medications, supporting the notion that conditional prescribing may be “lost in translation” across professional boundaries. The overall prevalence of PRN prescriptions in our study (24.5% of medication entries) is consistent with previously reported rates in geriatric settings, which range widely from 28% to over 90% depending on methodology and population [5,6,12]. As described in prior studies, PRN medications were predominantly gastrointestinal agents, analgesics, and psychotropic drugs. Indeed, the comparisons in our analysis support this observation, suggesting that the interpretation of PRN wording may vary according to the availability of objective clinical assessment criteria. In particular, psychotropic medications may be more challenging to assess than gastrointestinal agents or analgesics, for which standardized measures are often available. These therapeutic classes are frequently identified as high risk or potentially inappropriate in older adults due to their association with sedation, falls, delirium, constipation, and other adverse drug side effects [15]. Our findings reinforce concerns that PRN prescribing constitutes a significant proportion of medication exposure in hospitalized older adults and therefore represents a critical target for medication safety interventions.

### 4.2. Interpretation

A central finding of this study is the significant variability in how physicians, nurses, and pharmacists interpreted the wording of PRN prescriptions. Our findings demonstrate that substantial differences may exist between healthcare professionals when interpreting the same PRN prescription, highlighting interprofessional variability as a potentially underrecognized source of medication errors and communication failures in geriatric practice. With the exception of a limited number of intra-professional comparisons, nearly all pairwise comparisons produced statistically significant differences. This variability suggests that the clarity of PRN prescriptions is not objective and may depend on professional background, clinical experience, and contextual interpretation. Several factors may explain these interprofessional differences. Healthcare professionals differ in their training, clinical experience, professional responsibilities, and familiarity with medication management processes, all of which may influence how PRN prescriptions are interpreted. In addition, the healthcare professionals for whom PRN prescriptions are primarily intended may differ across care settings, potentially influencing how they are interpreted and implemented. Depending on local organization and workflows, PRN instructions may primarily be directed toward nurses, but may also involve physicians or other healthcare professionals, such as students or temporary staff. Unit-specific prescribing practices and organizational cultures may further shape expectations regarding the level of detail required in PRN wording. Taken together, these factors may contribute to differences in interpretation across both professional groups and care settings. Previous research has highlighted that nurses often bear primary responsibility for deciding whether to administer PRN medications, particularly psychotropics and analgesics [5,6,16]. When prescription wording lacks explicit operational criteria (e.g., measurable thresholds or standardized scales), administration decisions may rely heavily on inappropriate judgment. This variability supports the hypothesis that conditional prescribing relies heavily on non-standardized interpretation, particularly when wording lacks precise operational clinical criteria. Such discrepancies may translate into inconsistent medication administration practices and potentially increase the risk of medication-related harm in older hospitalized patients. This discrepancy between prescriber intent and administrator interpretation creates a potential gap in medication safety. In line with the prior literature, which emphasizes communication failures as a major contributor to medication errors [12,17], our findings suggest that ambiguous PRN wording may represent a structural vulnerability in the medication-use process.

In this sense, PRN prescriptions may indeed be “lost in translation” as what is intended by the prescriber may not be interpreted uniformly by other healthcare professionals, potentially leading to inconsistent administration, including overtreatment and undertreatment.

Multivariable analysis demonstrated that hospitalization in long-term care or rehabilitation units was independently associated with more appropriate PRN wording compared with acute geriatric units. Although no studies have compared the influence of hospitalization type on PRN medications, this may reflect more stable clinical conditions, longer lengths of stay, or more standardized prescribing practices in non-acute settings. Conversely, a higher total number of prescribed medications was associated with reduced appropriateness of PRN wording, in line with previous studies. Polypharmacy may also increase prescribing complexity, reducing the time physicians have for detailed documentation, and contributing to less precise conditional statements. This finding aligns with broader evidence linking polypharmacy to increased medication errors and adverse events in older adults. Importantly, previous studies have shown that several parameters, such as polypharmacy, length of stay, and hospitalization type, are predictive of PRN prescription use [18,19]. Our findings highlight that the wording of these prescriptions is essential for medication safety.

PRN prescribing is often perceived as a flexible and patient-centered strategy, enabling rapid symptom control [13]. However, flexibility without precision may compromise safety [12]. Given that 99% of patients received at least one PRN administration during the study period, the cumulative impact of ambiguous wording may be substantial at the population level. Standardization of PRN prescription wording—through structured templates, predefined clinical criteria (e.g., pain scores and agitation scales), or electronic prescribing prompts—could reduce interpretative variability. Interprofessional education and collaborative reviews of PRN prescriptions may further enhance shared understanding. The safety of PRN medicine and its management should therefore rely on a close collaboration and partnership between nurses, physicians, and pharmacists [8]. Our results reinforce the need to develop tools or strategies that ensure safe and appropriate PRN medicines management, such as protocols, PRN medication reviews, or good practice manuals, as described by Baker et al. Psychotropic PRN drugs should also be assessed in acute mental health wards [20]. In addition, Vaismoradi et al. argue that patients’ families and caregivers should always be involved in decisions to maximize their empowerment [21]. A 100% concordance was observed among healthcare professionals for protocolized PRN insulin prescriptions. Therefore, the positive association between protocolized PRN prescriptions and appropriate wording identified in the multivariate analysis supports the implementation of standardized prescribing practices and their extension, whenever feasible, to other pharmacological classes.

### 4.3. Limitations

This study has several strengths, including its prospective analysis of real-life prescriptions in a large geriatric hospital and the inclusion of independent, blinded assessments by three professional groups. The focus on interprofessional interpretation represents an original contribution to the literature on PRN prescribing. However, several limitations must be acknowledged. First, the study was conducted in a single center and relied on one-day assessments in each unit. As such, it may be subject to potential biases (such as seasonal effects, staffing levels, and the patient case-mix study design), which limit the generalizability of the findings. In addition, there was a marked imbalance in the number of patients across care settings, with a higher proportion in long-term care units. While this reflects real-world variability and distribution of hospital beds in various care settings, it may have affected comparability between units. Second, the present study focused on the appropriateness of wording in PRN prescriptions and interprofessional variability in their interpretation. However, we did not assess the actual administration of PRN medication. Therefore, although ambiguous PRN wording may contribute to inconsistent medication use, no causal relationship with adverse clinical outcomes or adverse drug events was assessed. Third, detailed characteristics of healthcare professionals, such as years of professional experience, clinical background, or specific training in medication management, were not collected and may have contributed to the observed variability in interpretation. Finally, this study initially focused only on prescriptions and did not examine patient characteristics; as such, we were unable to investigate the association between specific comorbidities or clinical parameters and PRN medication, as has been shown for age, dementia, length, and type of hospitalization [5,16]. This may also have affected perceptions of PRN.

## 5. Conclusions

PRN prescribing is common for hospitalized older adults. It is frequently characterized by variability in interpretation across healthcare professionals. In this context, PRN prescriptions may represent points of ambiguity in medication management when wording lacks precision and therapeutic intent risks being “lost in translation”. Improving clarity, standardization, and interprofessional understanding of PRN prescription conditions may enhance the consistency of medication-related decision-making in geriatric care. Further studies are needed to determine whether these differences in interpretation are associated with medication errors or adverse drug events.

## Figures and Tables

**Table 1:** Baseline characteristics of the prescriptions analyzed.

**Prescription Characteristics**	**n (%) or m (±sd)**			**N**
	AC ^1^	LTC ^2^	RC ^3^	
	34 (10.8%)	163 (51.6%)	119 (37.7%)	
Number of prescribed drugs	12.70 (4.74)	13.60 (5.28)	13.00 (4.44)	316
PRN ^4^ medication prescriptions	3.21 (1.77)	3.44 (1.86)	3.08 (1.37)	316
PRN medication entries	109 (25.2%)	560 (25.2%)	366 (23.6%)	4206

Results are expressed as n (%) or mean (standard type); N, number observed. ^1^ AC, acute care; ^2^ LTC, long-term care; ^3^ RC, rehabilitation care; ^4^ PRN, Pro Re Nata.

**Table 2:** Characteristics of PRN medication prescriptions.

**Prescription Characteristics**	**n (%) or m (±sd)**			**N**
	AC ^1^	LTC ^2^	RC ^3^	
	109 (10.5%)	560 (54.1%)	366 (35.4%)	1035
Type of PRN ^4^ drug				1035
Non-opioid analgesics	22 (20.2%)	136 (24.3%)	94 (25.7%)	
Opioid analgesics	13 (11.9%)	37 (6.61%)	38 (10.4%)	
Hypno-sedative drugs	20 (18.3%)	73 (13.0%)	51 (13.9%)	
Neuroleptics	15 (13.8%)	19 (3.39%)	9 (2.46%)	
Gastrointestinal medications	26 (23.9%)	224 (40.0%)	147 (40.2%)	
Insulin	5 (4.59%)	12 (2.14%)	10 (2.73%)	
Others	8 (7.34)	59 (10.5%)	17 (4.64%)	
PRN ^4^ with a defined end date				1035
Yes	0 (0.0%)	0 (0.0%)	0 (0.0%)	
No	109 (100%)	560 (100%)	366 (100%)	
Mean duration (days)	11.1 (9.94)	263 (274.0)	40.2 (46.4)	1035

Results are expressed as n (%) or mean (standard type); N, number observed. ^1^ AC, acute care; ^2^ LTC, long-term care; ^3^ RC, rehabilitation care; ^4^ PRN, Pro Re Nata.

**Table 3:** Assessment of PRN ^4^ prescription wording appropriateness.

Professional Group/Care Unit	Appropriate n (%)	Inappropriate n (%)	N
Nurse—AC ^1^	832 (80.4)	203 (19.6)	1035
Nurse—LTC ^2^	906 (87.5)	129 (12.5)	1035
Nurse—RC ^3^	828 (80.0)	207 (20.0)	1035
Physician—AC ^1^	931 (90.0)	104 (10.0)	1035
Physician—LTC ^2^	740 (71.5)	295 (28.5)	1035
Physician—RC ^3^	928 (89.7)	107 (10.3)	1035
Clinical pharmacist	674 (65.1)	361 (34.9)	1035
Overall healthcare professionals	531 (51.3)	504 (48.7)	1035

Results are expressed as n (%) or mean (standard type); N, number observed. ^1^ AC, acute care; ^2^ LTC, long-term care; ^3^ RC, rehabilitation care; ^4^ PRN, Pro Re Nata.

**Table 4:** Pairwise comparisons of appropriate PRN ^4^ prescription wording between professional groups and care settings (*p*-values).

Professional Group/Care Unit	Nurse—AC	Nurse—LTC	Nurse—RC	Physician—AC	Physician—LTC	Physician—RC	Clinical Pharmacist
Nurse—AC ^1^	-	<0.001	0.31	<0.001	<0.001	<0.001	<0.001
Nurse—LTC ^2^		-	0.022	<0.001	<0.001	<0.001	<0.001
Nurse—RC ^3^			-	<0.001	0.44	<0.001	<0.001
Physician—AC ^1^				-	<0.001	<0.001	<0.001
Physician—LTC ^2^					-	<0.001	<0.001
Physician—RC ^3^						-	<0.001
Clinical pharmacist							-

Results represent *p*-values from pairwise chi-square comparisons of PRN prescription appropriateness between professional groups and care settings. ^1^ AC, acute care; ^2^ LTC, long-term care; ^3^ RC, rehabilitation care; ^4^ PRN, Pro Re Nata.

**Table 5:** Factors associated with the appropriateness of PRN prescription wording.

**Covariates**	**Crude OR**	**CI95%**	***p*** **Value**
Care Unit			
AC ^1^	reference		
LTC ^2^	3.68	2.31–5.85	<0.001
RC ^3^	2.41	1.51–3.86	<0.001
Number of prescription entries (>5)	0.17	0.02–1.45	0.106
Number of prescription entries	0.98	0.96–1.00	0.030
Number of PRN ^4^ prescriptions (>2)	1.24	0.57–2.73	0.589
Number of PRN ^4^ prescriptions	0.88	0.83–0.94	<0.001
Number of prescriptions under a therapeutic protocol (>1)	1.80	1.19–2.76	0.006
Number of prescriptions under a therapeutic protocol	1.80	1.19–2.76	0.006
Non-opioid analgesic medications	0.73	0.55–0.98	0.036
Gastrointestinal medications	6.44	4.84–8.58	<0.001
Hypno-sedative medications	0.02	0.01–0.06	<0.001
**Covariates**	**Adjusted OR**	**CI95%**	***p*** **Value**
Care Unit			
AC ^1^	reference		
LTC ^2^	4.17	2.48–7.03	<0.001
RC ^3^	2.07	1.22–3.53	0.007
Number of prescriptions under a therapeutic protocol (>1)	2.27	1.39–3.63	0.001
Number of PRN ^4^ prescriptions	0.87	0.82–0.94	<0.001
Gastrointestinal medications	4.37	3.22–5.94	<0.001
Hypno-sedative medications	0.05	0.02–0.11	<0.001

Odds ratio (OR); 95% confidence interval (CI95%); and multivariate logistic regression model. AC ^1^, acute care; LTC ^2^, long-term care; RC ^3^, rehabilitation care; PRN ^4^, Pro Re Nata.

## Data Availability

Data is contained within the article or Supplementary Material. The original contributions presented in this study are included in the article/Supplementary Material. Further inquiries can be directed to the corresponding author.

## Supplementary Materials

The following supporting information can be downloaded at https://www.mdpi.com/article/10.3390/geriatrics11040103/s1, Table S1: Inter-rater agreement between nurses, physicians, and clinical pharmacists for the assessment of PRN prescription appropriateness across care settings.
