# Supplementary material for: Lost in Translation: Interprofessional Variability in Understanding Pro Re Nata (PRN) Prescribing in Geriatric Practice—A Prospective Observational Study
Source: Geriatrics (Basel). 2026 Aug 11;11(4):103. doi: 10.3390/geriatrics11040103 (PMC13512332; doi:10.3390/geriatrics11040103)
Supplement: Supplementary file 1 [file geriatrics-11-00103-s001.zip › geriatrics-4303424-supplementary.pdf]

Supplementary Table S1. Inter-rater agreement between nurses, physicians, and clinical pharmacists for the assessment of PRN prescription appropriateness across care settings

Care setting	Pairwise comparison	Cohen's k
**AC[1]**	Nurse – AC[1] / Physician – AC[1]	0.31
	Nurse – AC[1] / Clinical Pharmacist	0.40
	Physician – AC[1] / Clinical Pharmacist	0.17
	Overall agreement (Fleiss' k)	0.28
**RC[3]**	Nurse – RC[3]/ Physician – RC[3]	0.24
	Nurse – RC[3] / Clinical Pharmacist	0.49
	Physician – RC[3]/ Clinical Pharmacist	0.29
	Overall agreement (Fleiss' k)	0.33
**LTC[2]**	Nurse – LTC[2]/ Physician – LTC[2]	0.49
	Nurse – LTC[2] / Clinical Pharmacist	0.29
	Physician – LTC[2] / Clinical Pharmacist	0.36
	Overall agreement (Fleiss' k)	0.36

Cohen's k was used to assess pairwise agreement between professional groups, and Fleiss' k was used to assess overall agreement among nurses, physicians, and clinical pharmacists.
AC[1] Acute Care, LTC[2] Long-Term Care, PRN[4] Pro Re Nata, RC[3] Rehabilitation Care
